# PORCINE CYSTICERCOSIS RISKS: AWARENESS, ATTITUDES AND PERCEPTIONS ON SAFETY PRACTICES AMONG FARMERS, BUTCHER-OWNERS AND CONSUMERS IN WESTERN KENYA

**DOI:** 10.21010/ajid.v14i2.3

**Published:** 2020-07-31

**Authors:** Marie-Françoise Mwabonimana, Anthony Macharia King’ori, Charles Muleke Inyagwa, Bockline Omedo Bebe

**Affiliations:** 1 Department of Animal Sciences, Faculty of Agriculture, Egerton University, P.O. Box 536-20115, Egerton, Kenya; 2 College of Animal Sciences and Veterinary Medicine, University of Rwanda, P.O. Box 210, Musanze, Rwanda; 3 Department of Veterinary Medicine, Faculty of Veterinary Medicine and Surgery, Egerton University, P.O. Box 536 – 20115, Egerton, Kenya

**Keywords:** Pork, quality, safety, *T. solium;* porcine cysticercosis, value chain actors

## Abstract

**Background:**

The demand for pork is increasing in Africa with the increasing need for animal protein in the household diets. But pork safety and quality remains a pervasive concern that needs intervention to assure consumers of protection from Porcine Cysticercosis (PC) contamination. This study assessed among farmers, butcher-owners and consumers in Western Kenya about their awareness, attitudes and perceptions about safety practices regarding risk of PC.

**Materials and Methods:**

Data were obtained using structured questionnaires in cross-sectional survey interviews with 162 farmers, 26 butcher-owners and 92 consumers from Busia and Kakamega Counties. The data were in binary response, so were analyzed with Chi - square test.

**Results:**

Only two in ten farmers had knowledge of *Taenia solium* parasite (24.1%), risk factors in PC transmission (21.6%) and could associate pig management system with PC (17.3%). A larger proportion (p<0.01) of the butcher owners perceived pork from slaughter slabs (76.9%) and home slaughters (73.1%) as presenting high risks but considered pork from the butcheries (69.1%) and eateries (61.5%) as presenting no risks. Among the consumers, majority strongly agreed (p<0.05) that pork in the market (85.9%), from slaughter slabs (92.4%) and butchery (81.5%) was safe but a larger proportion strongly disagreed that pork from the eateries exposed them to cysticercosis (64.1%).

**Conclusion:**

The awareness about risks of PC was low among farmers. Butcher-owners and consumers perceived pork safety differently along the value chain. Strengthening public education about PC risks and pork safety among all actors in the pork value chain in Western Kenya is recommended.

## Introduction

The pork industry in Kenya is growing and is differentiated into specialized business units along the value, consisting of feed millers, producers, abattoirs, processors and retailers. The pork value chain is organized in a way that live pigs are sold at the farm gate, slaughtered pigs at the abattoirs, pork sold at the butcheries and processed products sold at specialized pork eateries (Levy *et al.*, 2013). In Western Kenya, smallholders dominate pork production but lack access to functionally good pig slaughterhouses to enhance pork safety for consumers (Levy, 2014). Improving pork safety and quality is important for consumers because in the food supply chain, the consumer level of trust depends on safety and quality associated with the product marketed (Taylor *et al.*, 2012).

Rapid growth in pork consumption should contribute (Bett *et al.*, 2012; FAO, 2012) to improved food security and nutrition because pork is rich in protein, Vitamin B12, iron and selenium, Vitamin C, niacin, phosphorus and zinc (USDA, 2018). However, consumption of pork may expose consumers (Davies, 2011) to hazards and risk of Porcine Cysticercosis (PC) *Taenia solium* in pigs, associated with PC, may infest people through food following ingestion of the parasite larval cysts in undercooked and contaminated pork (Saw *et al.*, 2015). Focus on the prevention, control and reduction of the hazard and risk to PC is thus a public health objective (Schär *et al.*, 2014; Inpankaew *et al.*, 2015). The public health objective promotes “one health concept” for PC eradication (WHO/TDR, 2012; Torgerson *et al.*, 2015; Schurer *et al.*, 2016).

Integrating public health education with control strategies could promote effective and sustainable reduction of the risk of PC infestation in humans (O’neal *et al.*, 2012, Thys *et al.*, 2016). Creating public awareness is an important component of “one health approach” involving human, veterinary, environmental and social sectors. Integrated into ‘One health concept’ is public health education in combination with other control strategies for effective and sustainable eradication of the risk of PC infestation in humans (Ngowi *et al.*, 2008; Sorvillo *et al.*, 2011). Knowledge of awareness, attitudes and perceptions of safety practices among farmers, animal health workers, butcher-owners and consumers in addressing risks of PC infestation is important for the control of *Taenia solium* (GALVmed, 2017; Kungu *et al.*, 2017). This study examined the extent of awareness, attitudes and perceptions on safety practices among farmers, butcher-owners and consumers in Western Kenya on the risk factors for PC.

## Materials and Methods

Farmers were randomly selected from local villages known for high concentration of pigs, slaughter slabs, pork butcheries and consumers. Structured questionnaires were administered to 280 respondents of which 162 were farmers, 26 were butcher-owners and 92 were consumers. Snowball sampling was adopted for the survey to reach the targeted study population. The questionnaire had binary responses at production, trade and consumer levels. The responses were on demographic characteristics, farmer awareness about pig management, and risk transmission factors for PC. Butcher-owners and consumers were interviewed on attitudes and perceptions on safety practices for pork in the market.

### Statistical analysis

Data collected was entered in Excel database, and thereafter exported to the Statistical Analysis System version 9.1.3 (SAS, 2006). The analysis was on frequency distribution with Chi-square test statistics to examine the relative differences in awareness, attitudes, and perceptions about safety practices.

## Results

### Demographic characteristic of respondents.

Out of the 162 farmers interviewed, 37.7% were aged between 21 and 30 years, 53.1 % were of the female gender, 41.7 % had no formal school education and 77.2% had kept pigs for a period of 6 to 10 years. Among the 26 butcher-owners interviewed, 53.9% were between 11-20 years old, 92.3% were males, and 57.7% had attained primary level education, while 46.2% had 1-5 years of experience in pork butcher trade. Of the 92 consumers interviewed, 48.9% were between 31-40 years of age, 83.7% were females and 46.7% had not acquired formal education.

### Farmer’s awareness of risk of Porcine Cysticercosis

Table 1 shows the frequency distribution of farmers by their awareness about the pig management systems, *Taenia solium* parasite and the possible risks factors of its transmission. The estimated frequencies among those interviewed showed that only about two-in-ten farmers declared being aware of the link between pig management system and PC compared (p<0.0001) to eight-in-ten (17.3% vs. 82.7%) who declared that they were not aware. Furthermore, results revealed that two-in-ten farmers declared that they were aware of the *Taenia solium* parasite compared (p<0.0001) to eight-in-ten (24.1% vs. 75.9%) who claimed not being aware (χ2 43.556^a^, *p*.value 0.0001). Regarding awareness about risk factors in the transmission of porcine cysticercosis, only two-in-ten farmers were aware compared (p<0.0001) to eight-in-ten (21.6% vs 78.4%) who claimed not being aware.

**Table 1:** Frequency distribution of farmers by their awareness of transmission factors for *T*.* solium* cysticercosis

Awareness of:	Response	Frequency	Percent	Chi-Square (χ2)	P-value
Knowledge about *Taenia solium* parasite				43.5565	0.0001
	Aware	39	24.1		
	Not aware	123	75.9		
Knowledge about the link between pigs management systems and PC				69.3580	0.0001
	Aware	28	17.3		
	Not aware	134	82.7		
Knowledge about risks for PC transmission				52.2469	0.0001
	Aware	35	21.6		
	Not aware	127	78.4		

### Butcher-owners and consumers’ attitudes towards safety of pork in the market

Table 2 presents the frequency distribution of butcher-owners’ attitudes to issues of safety of pork sold at different retail outlets along the value chain. While more (p<0.01) of the butcher owners interviewed had the perception that pork from slaughter slabs and home slaughters has high risks and pork from the butcheries has no risks, they could not (p>0.05) split on the safety of the pork from the eateries. For pork from slaughter slabs, about eight-in-ten of the butcher owners interviewed had the perception that risk was high compared to two-in-ten (76.9% vs. 23.1%) that had the perception that there are no risks. For pork from home slaughters, about seven-in-ten of the butcher owners interviewed had the perception that risk was high compared to three-in-ten (73.1% vs. 26.91%) that had the perception that there were no risks. In contrast, pork from the butcheries had about three-in-ten perceiving that risk was high compared to seven-in-ten (30.8% vs. 69.2%) that had the perception that there were no risks. Though the perception of risk being high or no risk was not statistically different (p>0.05) for pork from the eateries, fewer had the perception that risk is high (38.5% vs. 61.5%).

**Table 2:** Frequency distribution of Butcher-owners’ attitudes to safety of pork at different retail outlets

Pork sale point	Risk perception	Frequency	Percent	Chi-Square (χ^2^)	* P- value*
Home slaughter				5.5385	0.0186
	High risk	19	73.1		
	No risk	7	26.9		
Slaughter Slabs				7.5385	0.0060
	High risk	20	76.9		
	No risk	6	23.1		
Butchery				3.8462	0.0499
	High risk	8	30.8		
	No risk	18	69.2		
Eateries				1.3846	0.2393
	High risk	10	38.5		
	No risk	16	61.5		

Table 3 presents the frequency distribution of consumer perception to safety of pork in the market in response to whether they strongly agreed or disagreed with the specific statements put to them. More of the consumers interviewed strongly agreed (p<0.05) that pork was generally safe (85.9% vs. 14.1%), that pork from the slaughter slab was safer than pork from the farm (92.4% vs. 7.6%) and that pork from butcheries is generally safer than pork from the eateries (81.5% vs. 18.5%). However, more of the consumers (p<0.05) interviewed strongly disagreed that pork from the eateries exposed humans to cysticercosis (64.1% vs. 35.9%). On the other hand, consumers could not split (p>0.05) on whether undercooked pork was more likely to transmit cysticercosis to humans and whether they always cooked pork well before eating.

**Table 3:** Frequency distribution of consumer perception on safety of pork in the market

Perception	Agreement	Frequency	Percent	Chi-Square (χ^2^)	*P- value*
Pork sold is generally safe				47.3478	0.0001
	Strongly agree	79	85.9		
	Strongly disagree	13	14.1		
I always cook well the pork before eating				3.5217	0.0606
	Strongly agree	55	59.8		
	Strongly disagree	37	40.2		
Undercooked pork is more likely to transmit cysticercosis to human				1.5652	0.2109
	Strongly agree	52	56.5		
	Strongly disagree	40	43.5		
Pork from the slaughter slab is safer than pork from farm				66.1304	0.0001
	Strongly agree	85	92.4		
	Strongly disagree	7	7.6		
Pork from butchers is generally safer than pork from the eateries				36.5652	0.0001
	Strongly agree	75	81.5		
	Strongly disagree	17	18.5		
Pork from the eateries expose human to cysticercosis				7.3478	0.0067
	Strongly agree	33	35.9		
	Strongly disagree	59	64.1		

## Discussion

This study investigated awareness, attitudes and perceptions on safety practices among farmers, butcher-owners and consumers about the risk factors for PC in Western Kenya. Results showed only two-in-ten farmers had knowledge of *Taenia solium* parasite, risk factors in PC transmission and could associate pig management system with PC. These findings differed with those of Adenuga *et al.*, (2018) who found high level of awareness among farmers, with seven-in-ten (70.5%) being aware of porcine cysticercosis and about half (47.8%) knowing about its transmission as a zoonotic disease. Mishra *et al.*, 2007 reported high level of awareness among farmers (59.1%) but with low awareness about pork tapeworm transmission (35.0%). Results of the present study may have differed from those others because it was carried out in the rural area of Western Kenya where pig keeping had become a popular small-holder activity for low-income families where over half (57.6%) lived in poverty. These resource-poor families engage in pig production using the traditional scavenging feeding system because of inability to invest in modern housing, commercial feeds and herd health programme. Pig production is a diversification livelihood strategy and not a major source of income streams for these farmers. About half (50%) of the farmers were without formal school education to enable them be trained by extension staff on modern pig husbandry (Nantima *et al*., 2015a, Kithinji *et al.*, 2017) as compared to pig farmers in Botswana and Tanzania where 15 to 25% of farmers had secondary education (Nsoso *et al.*, 2006; Karimuribo *et al.*, 2011).

The results of this study concur with the findings of Sibongiseni *et al.*, (2016) and Mwendia *et al.*, (2018) which reported an association between poor knowledge of *T. solium* infections and poor hygiene by farmers. This practice was common in Western Kenya where farmers owned and used dilapidated, unhygienic latrines for human waste disposal. Therefore, this study suggests the need for farmers to be trained on the three variables namely, pig management systems, *T. solium*, porcine cysticercosis as important tools for control in the two areas.

Results of this study showed that the risks of pork in the market was perceived to be high at slaughter slabs and home slaughters, and no risks at the butchery and eateries. Findings elsewhere (Ocaido *et al.*, 2013; Fahrion *et al.*, 2014; Fogang *et al.*, 2015), reported that pork from informal market was riskier to human health exposing consumers to zoonotic diseases. The findings of this study are in agreement with reports of Ngasala *et al.*, (2015) and Gayatri *et al.*, (2017) who reported that butcher-owners as knowledgeable people able to protect human beings through disease prevention and control. These results suggested` the need for creation of awareness of the risk of disease transmission to butcher-owners along the pork value chain in the study area.

Results revealed that the majority of consumers agree with the perceptions, all except for the eateries where consumers were less likely to agree in strong agreement about exposure to cysticercosis in the eateries. The present results confirm the findings by Kagira *et al.*, (2010) and FAO, (2012) who reported that most butcheries in Western Kenya had restaurants/eateries attached where pork was sold cooked (41%). The butcheries sold also raw pork (59%). Studies in Burkina Faso (Ngowi *et al.*, 2017), reported that, boiling was the common traditional method for cooking pork. Boiling of pork products exposes consumers to *Taenia solium* (Thomas, 2014; Cook, 2015) due to undercooking since consumers do enjoy and prefer the juiciness of the meat (Levy *et al.*, 2014). A study by Nguhiu *et al.*, (2020) in Thika sub-County of Kiambu County of Kenya, reported the frying as the preference method by consumers. This method may expose humans as it cannot kill all cysts. This expresses how consumers ignored the fact that pork from the eateries could expose humans to cysticercosis due to the practice of eating undercooked pork. It is also suggesting that health education could significantly increase knowledge and awareness of the disease, and can inspire behavioral change that will reduce disease transmission through thorough cooking practices. Consumers were not sure of the safety aspect of the meat consumed in the vibandas because of doubts of health inspection and meat source. Our findings confirmed those by Mutua *et al.*, (2019) on the traceability approach for consumers along the traditional pork value chain in western Kenya. Hence, our results suggest the need for consumers to be educated on the pork eating habits as it predisposes humans to the utter risk of *T. solium* infection in the study area.

## Conclusion

The study concluded that farmers from Western Kenya had little knowledge of pig management, *Taenia* parasite or its transmission. Results also indicate that butcher-owners and consumers hold different views about where safe pork is found in the market. Therefore, public education about PC risks and pork safety is necessary among all stakeholders in the pork value chain in Western Kenya. This should involve training pig farmers, pork consumers, butcheries to create awareness on transmission risk factors and strategies for the control of porcine cysticercosis. It should be possible to complement training with public education to stop consumer habits of eating undercooked pork from untraceable slaughter sources along the pork value chain, especially so in the local eateries. This predisposes humans to the risk of *T. solium* infection resulting in intestinal tapeworm when the pork eaten contains larval cysts. The stakeholders need to embrace multi-sector one health approach to break the *T. solium* life cycle. The main components of this campaign are recruitment of qualified pork inspectors and enforcement of meat inspection practices as requisite to the control of PC in Busia and Kakamega Counties.

**List of Abbreviations:**
CESAAM: : African Centre of Excellence in Sustainable Agriculture and Agribusiness Management
FAO: : Food and Agriculture Organization
GALVmed: : Global Alliance for Livestock Veterinary Medicines
OIE: : World Organisation for Animal Health
PC: : Porcine Cysticercosis
SAS: : Statistical Analysis System
*T. solium*: : *Taenia solium*
TDR: : Special Program for Research and Training in Tropical Diseases
USDA: : United States Department of Agriculture
Vs.: : Versus
WHO: : World Health Organization
χ^2^: _=_ Chi-square.

## References

[1] Adenuga A, Mateus A, Ty C, Borin K, Holl D, Sand S, Duggan V, Clark M, Smithe G.J.D, Coker R, Andrew V, Rudge J.W (2018). Seroprevalence and awareness of porcine cysticercosis across different pig production systems in south-central Cambodia. Parasite Epidemiology and Control.

[2] Bett H.K, Musyoka M.P, Peters K.J, Bolemann W (2012). Demand for Meat in the Rural and Urban Areas of Kenya:A Focus on the Indigenous Chicken. Economics Research International.

[3] Cook E. A. J (2015). Epidemiology of zoonoses in slaughterhouse workers in western Kenya. Thesis.

[4] Davies P.R (2011). Intensive swine production and pork safety. Foodborne Pathogens Disease.

[5] Fahrion A. S, Jamir L, Richa K, Begum S, Rutsa V, Ao S, Padmakumar V.P, Deka R.P, &Grace D (2014). Food-safety hazards in the pork chain in Nagaland, North East India:implications for human health. International journal of environmental research and public health.

[6] FAO (2012). Pig Sector Kenya. FAO Animal Production and Health Livestock Country Reviews. No. 3. Rome.

[7] Fogang Y. F, Savadogo A. A, Camara M, Toffa D. H, Basse A, Sow A. D, Ndiaye M. M (2015). Managing neurocysticercosis:Challenges and solutions. International Journal of General Medicine.

[8] Gayatri Khanal, &Sunita Poudel (2017). Factors Associated with Meat Safety Knowledge and Practices Among Butchers of Ratnanagar Municipality, Chitwan, Nepal:A Cross-sectional Study. Asia Pacific Journal of Public Health.

[9] GALVmed (2017).

[10] Inpankaew T, Murrell K.D, Pinyopanuwat N, Chhoun C, Khov. K. Sem, T, Sorn S, Muth S, &Dalsgaard A (2015). A survey for potentially zoonotic gastrointestinal parasites of dogs and pigs in Cambodia. Acta Parasitol.

[11] Kagira JM, Kanyari N, Maingi N, Githigia SM, Ng'ang'a JC, &Karuga JW (2010). Characteristics of the smallholder free range pig production system in Western Kenya. Trop Anim Health Prod.

[12] Karimuribo E.D, Chenyambuga S.W, Makene V.W, &Mathias S (2011). Characteristics and production constraints of rural-based small-scale pig farming in Iringa region, Tanzania. Livestock Research for Rural Development.

[13] Kithinji R.K, Kanui T.I, Ndathi J.N.A, &Mwobobia R.M (2017). Characterization of Pig Production Systems in Embu West Sub County, Embu County, Kenya. Int. J. Adv. Res.

[14] Kungu J. M, Dione M. M, Ejobi F, Ocaido M, &Grace D (2017). Risk factors, perceptions and practices associated with *Taenia solium* cysticercosis and its control in the smallholder pig production systems in Uganda:a cross-sectional survey. BMC Infectious Diseases.

[15] Levy M.A, Dewey C.E, Poljak Z, Weersink A, &Mutua F.K (2014). Comparing the operations and challenges of pig butchers in rural and peri-urban settings of western Kenya. African Journal of Agricultural Research.

[16] Levy M. A, Dewey C. E, Weersink A, Mutua F. K, &Poljak Z (2013). Pig marketing and factors associated with prices and margins in Western Kenya. Journal of Agricultural Economics and Development.

[17] Mishra D, Kalra V, &Aggarwal K (2007). Awareness about taeniasis and neurocysticercosis among municipal schoolteachers in Delhi. Journal of Communicable Diseases.

[18] Mutua F, Lindahl J, &Randolph D (2019). Possibilities of establishing a smallholder pig identification and traceability system in Kenya. Tropical Animal Health and Production.

[19] Mwendia S, &Notenbaert An (2018). Review of livestock production in Kakamega, Busia and Bungoma Counties in Western Kenya. International Center for Tropical Agriculture (CIAT).

[20] Nantima N, Ocaido M, Davies J, Dione M. M, Okoth E, Mugisha A, &Bishop R (2015a). Characterization of smallholder pig production systems in four districts along the Uganda-Kenya border. Livestock Research for Rural Development.

[21] Ngasala J.U, Nonga H.E, &Mtambo M.M (2015). Assessment of raw milk quality and stakeholders'awareness on milk-borne health risks in Arusha City and Meru District, Tanzania. Tropical Animal Health Production.

[22] Ngowi H, Ozbolt I, Millogo A, Dermauw V, Télesphore Somé T, Spicer P, Jervis L.L, Ganaba R, Gabriel S, Pierre Dorny P, &Carabin H (2017). Development of a health education intervention strategy using an implementation research method to control taeniasis and cysticercosis in Burkina Faso. Infectious Diseases of Poverty.

[23] Ngowi H. A, Carabin H, Kassuku A. A, Mlozi M. R. S, Mlangwa J. E. D, &Willingham III A. L (2008). A health-education intervention trial to reduce porcine cysticercosis in Mbulu District, Tanzania. Preventive Veterinary Medicine.

[24] Nguhiu P, Kabuage L, Warutere P, Kinyua K, &Kanina P (2020). Emergence of Cysticercosis, a neglected meat-borne notifiable zoonosis in Thika sub County of Kiambu County, Kenya. African Journal of Rural Development.

[25] Nsoso S. J, Mannathoko G. G, &Modise K (2006). Monitoring production, health and marketing of indigenous Tswana pigs in Ramotswa village of Botswana. Livestock Research for Rural Development.

[26] O'neal S. E, Townes J. M, Wilkins P. P, Noh J. C, Lee D, Rodriguez S, Garcia H. C, Stauffer W. M (2012). Seroprevalence of antibodies against Taenia solium cysticerci among refugees resettled in United States. Emerging Infectious Diseases.

[27] Ocaido M, Kristina R, &Delia G, O'neal S. E, Townes J. M, Wilkins P. P, Noh J. C, Lee D, Rodriguez S, Garcia H. C, Stauffer W. M (2013). Food safety and zoonotic hazards in pig value chains in East Africa. In Africa ecosante/ecohealth. 1–15. Emerging Infectious Diseases.

[28] SAS Institute Inc (2006).

[29] Saw Bawm, &Lat Lat Htun (2015). Parasitic Zoonoses in Livestock and Domestic Animals of Myanmar and Neighbouring Countries. Asian Journal of Animal and Veterinary Advances.

[30] Schär F, Inpankaew T, Traub R.J, Khieu V, Dalsgaard A, Chimnoi W, Chhoun C, Sok D, Marti H, Muth S, &Odermatt P (2014). The prevalence and diversity of intestinal parasitic infections in humans and domestic animals in a rural Cambodian village. Parasitol Int.

[31] Schurer J.M, Mosites E, Meschke C.Li.S, &Rabinowitz P.R, Sorvillo F, Wilkins P, Shafir S, Eberhard M (2016). Community-based surveillance of zoonotic parasites in 'One Health'world:a systematic revieuw. One Health, 2, 166-174. https://doi.org/10.1016/j.onehlt.2016.11.002. Public health implications of cysticercosis acquired in the United States. Emerging infectious diseases.

[32] Sibongiseni T.G, Oguttu J.W, &Masafu M.M (2016). Pig farming in rural South Africa:A case study of uThukela District in KwaZulu-Natal. Indian Journal of Animal Research.

[33] Sorvillo F, Wilkins P, Shafir S, &Eberhard M (2011). Public health implications of cysticercosis acquired in the United States. Emerging infectious diseases.

[34] Taylor A, Coveney J, Ward P. R, Dal Grande E, Mamerow L, Henderson J, &Meyer S. B (2012). The Australian Food and Trust Survey:Demographic indicators associated with food safety and quality concerns. Food Control.

[35] Thomas L (2014). Epidemiology of Taenia solium Cysticercosis in western Kenya. Thesis.

[36] Thys S, Mwape K. E, Lefèvre P, Dorny P, Phiri A, Marcotty T, Phiri IK, Gabriël S (2016). Why pigs are free-roaming:communities'perceptions, knowledge and practices regarding pig management and taeniosis/cysticercosis in a Taenia solium endemic rural area in Eastern Zambia. Veterinary parasitology.

[37] Torgerson P.R, Devleesschauwer B, Praet N, Speybroeck N, Willingham A.L, Kasuga F, Bokni M.B, Zhou XN, Fevre E.M, Sripa B, Gargouri N, Furst T, Budke C.M, Carabin H, Kirk M.D, Angulo F.J, Hayelaar A, &Silva N (2015). World Health Organization Estimates of the Global and Regional Disease Burden of 11 Foodborne Parasitic Diseases, 2010:A Data Synthesis. PLoS Med.

[38] USDA (United States Department of Agriculture) (2018). NutritionData.com [WWW Document]. Natl. Nutr. Database.

[39] WHO/TDR Disease Reference Group on Zoonoses and Marginalized Infectious Diseases of Poverty (2012). Research priorities for zoonoses and marginalized infections.

